# Phycoerythrin from *Colaconema* sp. Has Immunostimulatory Effects on the Whiteleg Shrimp *Litopenaeus vannamei* and Increases Resistance to *Vibrio parahaemolyticus* and White Spot Syndrome Virus

**DOI:** 10.3390/ani11082371

**Published:** 2021-08-11

**Authors:** Po-Tsang Lee, Jing Huang, Chin-Yi Huang, Zi-Xuan Liu, Han-Yang Yeh, Huai-Ting Huang, Li-Li Chen, Fan-Hua Nan, Meng-Chou Lee

**Affiliations:** 1 Department of Aquaculture, National Taiwan Ocean University, Keelung City 20224, Taiwan; leepotsang@mail.ntou.edu.tw (P.-T.L.); haungjim31336@gmail.com (J.H.); thormars@gmail.com (C.-Y.H.); lzxuan08@gmail.com (Z.-X.L.); 20833001@mail.ntou.edu.tw (H.-Y.Y.); twinkleqazwsx784@gmail.com (H.-T.H.); fhnan@mail.ntou.edu.tw (F.-H.N.); 2 Institute of Marine Biology, National Taiwan Ocean University, Keelung City 20224, Taiwan; joechen@ntou.edu.tw; 3 Center of Excellence for the Oceans, National Taiwan Ocean University, Keelung City 20224, Taiwan; 4 Center of Excellence for Ocean Engineering, National Taiwan Ocean University, Keelung City 20224, Taiwan

**Keywords:** phycoerythrin, shrimp, innate immunity, vibrio, WSSV

## Abstract

**Simple Summary:**

In this study, we found that phycoerythrin from *Colaconema* sp. can differentially stimulate the immune response of whiteleg shrimp in vitro and in vivo and could potentially be used as an immunomodulator in shrimp culture.

**Abstract:**

We investigated whether phycoerythrin (PE), a pigment sourced from marine algae, could act as an immunomodulatory agent in whiteleg shrimp (*Litopenaeus vannamei*). To this end, PE was extracted and purified from a PE-rich macroalgae, *Colaconema* sp. Our in vitro analysis demonstrated that PE enhanced prophenoloxidase and phagocytosis activity but inhibited the production of reactive oxygen species in hemocytes. Additionally, the PE signal could be detected using an in vivo imaging system after its injection into the ventral sinus of the cephalothorax of whiteleg shrimp. The expression profiles of fourteen immune-related genes were monitored in hemocytes from whiteleg shrimp injected with 0.30 μg of PE per gram of body weight, and crustin, lysozyme, penaiedin 4, and anti-lipopolysaccharide factor showed up-regulated post-stimulation. The induction of immune genes and enhancement of innate immune parameters by PE may explain the higher survival rates for shrimp that received different doses of PE prior to being challenged with *Vibrio parahaemolyticus* or white spot syndrome virus compared to controls. Combined, these results show that PE from *Colaconema* sp. can differentially stimulate the immune response of whiteleg shrimp in vitro and in vivo and could potentially be used as an immunomodulator in shrimp culture.

## 1. Introduction

Due to global improvements in the quality of life, the international demand for shrimp has been increasing. In 2019, the estimated global production of cultured marine shrimp was 4.45 million metric tonnes. However, global shrimp production in 2020 is predicted to have been severely reduced due to the COVID-19 pandemic [1]. *Litopenaeus vannamei*, also known as whiteleg shrimp, is one of the most important cultured shrimp species worldwide. *L. vannamei* is described as having a wide range of salt tolerance, rapid growth, and a high survival rate, as well as being well suited for artificial reproduction and high-density culture, and has thus become the main cultured shrimp species in Taiwan [2]. In 2016, the production of *L. vannamei* was 4.15 million metric tons, which constituted more than 80% of the total shrimp production [3]. However, the rapid growth of comprehensive and industrialized shrimp culture systems has been negatively affected by the incidence and dissemination of various infectious diseases, such as vibriosis and viral diseases.

Vibriosis is attributable to the Gram–negative bacterium *Vibrio* spp. [4,5] and is a great obstacle for whiteleg shrimp aquaculture, as infections result in high mortality in farming operations. There are several *Vibrio* species that have been identified as infectious agents in penaeid shrimp, such as *Vibrio harveyi*, *V. parahaemolyticus*, and *V. alginolyticus,* which form a majority of the bacterial pathogens that cause severe losses, particularly in hatcheries [6,7,8]. Additionally, approximately 20 viruses have been reported in shrimp culture. Among these viruses, the white spot syndrome virus (WSSV) has had a considerable impact on shrimp culture and, at present, still causes a serious disease problem [9]. WSSV is reported to spread via contaminated water and cannibalism of infected shrimp [10]. Therefore, the improvement of immunity and disease resistance in whiteleg shrimp has become crucial for the continuous growth of the shrimp aquaculture industry [11].

Seaweeds are rich in proteins, vitamins, carbohydrates, fibers, lipids, and minerals. Moreover, seaweeds have abundant polysaccharides, such as fucoidan, lipopolysaccharide, peptidoglycans, sodium alginate, and glucan [12,13]. Previous investigations have focused on the impact of these substances on reducing the mortality caused by microbes [14,15], improving immune system function [12,16], and increasing the resistance to ammonia stress in shrimp [17]. However, the effects of other components in macroalgae on aquatic animals have rarely been studied. Red algae possess pigments such as phycoerythrin (PE), phycocyanin (PC), and allophycocyanin (APC), along with other pigments such as chlorophyll a, beta–carotene, and a number of unique xanthophylls [18,19]. PE is considered a recently evolved phycobiliprotein, broadening the cyanobacterial photon absorbance ability toward the blue end of the visible solar spectrum [20]. PE consists of α and β subunits that contain 160 to 180 amino acids [21,22]. These subunits have a covalently attached open-chain tetrapyrrole chromophore, the so-called phycoerythrobilin [20]. In a few investigations, PE purified from the marine cyanobacterium *Phormidium* sp. was noted to exhibit antioxidant property against various reactive oxygen species (ROS) [23]. Additionally, PE can extend life expectancy and improve the health of *Caenorhabditis elegans* [24]. It has been speculated that PE displays life-prolonging and health-promoting effects due to its antioxidant properties [25,26]. To our knowledge, the applications of PE extracts in aquatic animals have not yet been explored. The present study, therefore, aims to investigate the effects of PE extracted from *Colaconema* sp. on whiteleg shrimp.

## 2. Materials and Methods

### 2.1. Preparation of Phycoerythrin

One gram of frozen algae was diluted in 900 µL of distilled water (obtained a from greenhouse, Department of Aquaculture, National Taiwan Ocean University). Then, 0.1 g quartz sand and 1 mm grinding beads were added to the alga-water mixture and homogenized by a FastPrep-24 homogenizer (TeenPrep, 6 cycles, 4.0 m∙s^−1^ for 5 s; MP Biomedicals, Solon, OH, USA). The homogenized mixture was centrifuged at 10,000× *g* at 4 °C for 10 min. The supernatant was collected and transferred to a beaker containing a stir bar and placed on a magnetic stirrer. While the sample was stirring, saturated ammonium sulfate was slowly added to bring the final concentration to 35% saturation. Once the total volume of ammonium sulfate was added, the beaker was moved to 4 °C and incubated overnight. The mixture was then transferred to conical tubes and centrifuged at 10,000× *g* for 10 min (Hitachi CR 21G; Hitachi Ltd., Tokyo, Japan). The supernatant was carefully collected and transferred to a beaker containing a stir bar and placed on a magnetic stirrer. While the sample was stirring, saturated ammonium sulfate was slowly added to bring the final concentration to 50% saturation, and then the beaker was again moved to 4 °C overnight. The mixture was then centrifuged at 10,000× *g* for 10 min. The supernatant was carefully removed and discarded, and the conical tube was well-drained. The pellet was resuspended in 10–30% of the starting volume in 1 × phosphate-buffered saline (PBS; pH 7.0). The resulting PE solution was transferred to dialysis tubing and dialyzed versus three changes of 1 × low salinity PBS.

The PE fraction was loaded on a HiPrep Sephacryl S-300 HR column (GE Healthcare, Chicago, IL, USA), and the loading sample was about 10% of the column volume. The gel filtration was developed with PBS containing 10 mM NaCl. The flow rate was set at 0.5 mL per min using a high-pressure peristaltic pump (Ecom LCP4100; Ecom, CZ, Czech Republic) and a UV detector (Ecom LCD 2084; Ecom, CZ, Czech Republic) connected to the rear of the column. The absorption spectra of the phycocyanobilin chromophore were measured on a UV-Vis spectrophotometer (Jasco Spectrophotometer V-630, Jasco, Tokyo, Japan) at the wavelengths 280, 498, and 565 nm. The eluate from the liquid chromatograph (Panchum FC-201 Del, Kaohsiung city, Taiwan) was collected after the separation of the different phycobiliproteins. The eluted solution was passed through a 0.22 µm filter (Merck Millipore, MA, Burlington).

Chromatographic analysis was performed using a high-performance liquid chromatography (HPLC) system (Thermo Fisher Scientific, Waltham, MA, USA). The separation was performed using a SUPREMA Linear M chromatographic column (5 μm, 8 mm × 300 mm, PSS) at 40 °C. The mobile phase was made of 0.05% (*v*/*v*) sodium azide in water with a flow rate of 1.0 mL per min. The column effluent was monitored at 545 nm. The CS-PE was serially diluted (2, 4, 8, 16, 32, 64, 128, and 256 times) and the UV absorbance values were used to calculate the phycobiliprotein levels. Fluorescence values were also determined using an excitation wavelength of 485 nm with emission measured at 590 nm using a fluorescence-cooled luminescence analyzer (Plate Chameleon; Hidex, Turku, Finland) to establish the content and fluorescence of the eluting solution, then further compared with the standard PE FL52412 (Sigma-Aldrich, St. Louis, MO, USA). The phycobiliprotein concentration was calculated according to the following equations:

PE (mg/mL) = (A_562_ − 2.8(PC) − 0.34(APC))/12.7

PC (mg/mL) = (A_615_ − 0.7(A_652_))/7.38

APC (mg/mL) = (A_652_ − 0.19(A_615_))/5.65

The purity index was measured using chromatography gel filtration and calculated using the formula:

Purity Index = A_562_/A_280_

### 2.2. Native (PAGE) and Sodium Dodecyl Sulfate (SDS)-Polyacrylamide Gel Electrophoresis (PAGE) Analysis

For the native PAGE, the soluble protein concentrations were first analyzed using the Bradford method to quantify the protein concentrations in the samples. Samples (5 μg), including the PE purified from this study and commercially available PE (Sigma-Aldrich, St. Louis, MO, USA), were diluted with sample buffer (62.5 mM Tris-HCl, pH 6.8, 40% glycerol, and 0.01% bromophenol blue) in a 1:1 ratio and separated via electrophoresis at conditions of 5% pyrochlore colloid and 8% dissociation gel, at 75 V and 50 mA. The gel was fixed with fixative (secondary water: methanol: glacial acetic acid = 1:4:5) and stained with 0.1% Coomassie brilliant blue G-250 and was subsequently decolored with the decolorizing solution.

For the SDS-PAGE, quantitative samples (5 μg) were mixed 1:1 with sample buffer (0.125 M Tris-HCl (pH 6.8), 4% SDS, 20% glycerol, 0.01% bromophenol blue, and 10% B-mercaptoethanol) and heated at 100 °C for 10 min. The heated sample was injected along with 5 μL of protein standard (Bio-Rad, Hercules, CA, USA) into a film consisting of 5% pyrochlore colloid and 15% dissociation gel for protein composition analysis. Electrophoresis conditions were 5% pyrochlore colloid and 8% dissociation gel, at 75 V and 50 mA. The gel was fixed with fixative (secondary water: methanol: glacial acetic acid = 1:4:5), stained with 0.1% Coomassie brilliant blue G-250, and decolored with the decolorizing solution.

### 2.3. Experimental Shrimp

Approximately 200 whiteleg shrimps (~7 g) were transported from a local farm (Yilan County, Taiwan) to the laboratory within 2 h by a fish transport truck and allocated to fiberglass circular tanks (1 m^3^) containing clean aerated water. The whiteleg shrimp were acclimated for 14 days at 27 ± 1 °C and were fed with a commercial diet (Tairoun Products Company Ltd., Taiwan) twice a day at 5% of their total body weight. The water quality parameters such as temperature (25–29 °C), pH (7.8–8.1), salinity (33–35‰), and dissolved oxygen (5.0–6.0 mg/L) were monitored daily. Additionally, 20% of culture water was changed daily with fresh clean seawater to maintain the concentrations of ammonia-*N* and nitrite-*N*, detected using a water testing kit (Kyoritsu chemical-check Laboratory Corporation), lower than 0.05 and 0.03 mg/L, respectively.

### 2.4. In Vitro Effects of PE on the Immune Response of Hemocytes

#### 2.4.1. Prophenoloxidase Activity (PO)

Hemolymph withdrawn from ten whiteleg shrimp (12.0 ± 1.5 g) was mixed with ice-cold anticoagulant buffer (AC, 450 mM NaCl, 10 mM KCl, 10 mM HEPES, 10 mM EDTA-Na_2_, pH 7.3) at a ratio of 1:9, followed by centrifugation to remove the supernatant. Cells (1 × 10^6^ cells/mL) were incubated for 1 h with 0 (Control), 8 (T1), 14 (T2), 27 (T3), or 52 (T4) μg/mL PE at ambient temperature. Subsequently, cells were centrifuged at 800× *g* for 10 min at 4 °C and sonicated for 5 s four times. A 50 μL volume of cell suspension was inoculated into a 96-well plate and added with 50 μL of a 3 mg/mL l-dihydroxyphenylalanine solution (L-DOPA, Sigma-Aldrich, St. Louis, MO, USA). PO activity was measured after a 15 min incubation at room temperature using a microplate reader (BioTek, Winooski, VT, USA) at 490 nm [27,28].

#### 2.4.2. Reactive Oxygen Species (ROS) Production

Production of ROS was measured according to the published methods [29] with a minor modification. In short, hemocytes (1 × 10^6^ cells/mL) in modified complete Hank’s balanced salt solution (MCHBSS; 10 mM calcium chloride, 3 mM magnesium chloride, 5 mM magnesium sulfate in HBSS (Gibco, Carlsbad, CA, USA)) were incubated with 0 (Control), 8 (T1), 14 (T2), 27 (T3), or 52 (T4) μg/mL PE for 30 min at room temperature in a 96-well plate. The plate was then centrifuged at 800× *g* for 20 min at 4 °C and the supernatant was discarded. Subsequently, 100 μL of 2′,7′-Dichlorofluoresceindiacetate (DCFH-DA, final concentration of 0.1 mM; Sigma-Aldrich, St. Louis, MO, USA) was added into each well and cells were incubated in the dark for 30 min. Then, cells were washed twice with whiteleg shrimp salt solution (SSS) buffer (450 mM NaCl, 10 mM KCl, 10 mM HEPES, pH 7.3). A spectrofluorometer (Synergy H1 Hybrid Multi-Mode Reader; BioTek, Winooski, VT, USA) with an excitation wavelength of 480 nm and an emission wavelength of 523 nm was used to measure the fluorescence units (RFU).

#### 2.4.3. Phagocytosis Rate

The phagocytosis assay was performed according to published methods [30,31]. Diluted hemolymph (1000 μL, 1 × 10^6^ cells/mL) in MCHBSS was incubated with 0 (Control), 8 (T1), 14 (T2), 27 (T3), or 52 (T4) μg/mL PE for 30 min at room temperature. Subsequently, latex beads (carboxylate-modified polystyrene fluorescent yellow-green L4655 Ø = 1.0 μm, Sigma-Aldrich, St. Louis, MO, USA) were added into tubes at a rate of 100 beads per hemocyte and left in the dark for 2 h. Non-engulfed beads were washed off, and cells were rinsed two times with trypan blue (1.2 mg/mL) [32] before the addition of 1000 μL of AC. The phagocytosis rate was examined using BD FACSAria III Flow cytometry (BD Biosciences, Franklin Lakes, NJ, USA).

### 2.5. Gene Expression Analysis by Quantitative Real-Time Polymerase Chain Reaction (qRT-PCR)

Twenty healthy whiteleg shrimp (body weight 16.08 ± 0.09 g) were injected with 20 μL of SSS buffer alone (*n* = 10) or SSS buffer containing PE to achieve doses of 0.30 μg/g body weight (*n* = 10) and whiteleg shrimps were maintained at 28 °C in aquariums during the period of observation. The whiteleg shrimp hemocyte total RNA extraction method was modified from a previous study [33]. Briefly, 500 μL of hemocyte was taken at 8 and 24 h post-injection from 5 whiteleg shrimps. One part of the hemocyte was mixed with one part of AC prior to centrifugation at 800× *g* for 10 min. Cells were lysed using 500 μL of Trizol Reagent (Sigma-Aldrich, St. Louis, MO, USA) and mixed with 100 μL of chloroform. The tubes were vortexed for 10 s, incubated at ambient temperature for 3 min, and centrifuged at 13,000× *g* for 20 min at 4 °C. The colorless upper aqueous phase was combined with an equal volume of isopropanol (Sigma-Aldrich, St. Louis, MO, USA) followed by a 10 min incubation at ambient temperature. Samples were then centrifuged at 13,000× *g* for 10 min at 4 °C. The supernatant was discarded and the precipitated RNA pellet was washed with 1 mL of 75% ethanol twice and air-dried. Twenty microliters of nuclease-free water (Thermo Fisher Scientific, Waltham, MA, USA) was then added into each tube to dissolve the RNA pellet. Total RNA was subjected to a SpectraMax QuickDrop Micro-Volume Spectrophotometer (Molecular Devices, San Jose, CA, USA) for quality and quantity inspection. To avoid genomic DNA (gDNA) contamination, RNA samples were treated with DNase using the iScript gDNA Clear cDNA Synthesis Kit (Bio-Rad, Hercules, CA, USA) following the manufacturer’s instructions. Subsequently, DNase-treated RNA was reverse transcribed using the iScript Reverse Transcription Supermix. The reaction program was set as 25 °C for 5 min, followed by a 20 min incubation at 46 °C. Finally, the reaction was terminated at 95 °C for 1 min. cDNA samples were then diluted 10 times with nuclease-free water.

The expression of elongation factor-1α (*EF-1α*), lysozyme (*Lyz*), superoxide dismutase (*SOD*), glutathione peroxidase (*GPO*), *proPO I*, *proPO II*, penaeidin 2–4 (*Pen2*, *Pen3* and *Pen4*), peroxinectin (*PX*), alpha-2-macroglobulin (*A2M*), crustin (*Cru*), and clotting protein (*CP*) genes were detected using the primers listed in Table 1. The qPCR reactions (20 μL) consisted of 4 μL cDNA, 10 μL of RealQ Plus 2 × Master Mix (Ampliqon A/S, Odense M, Odense, Denmark), 2 μL primer (1 μL each for forward and reverse primer), and 4 μL of nuclease-free water. The qPCR was performed using a StepOnePlus Real-Time PCR machine (Thermo Fisher Scientific). The cycling protocol was one cycle of initial denature at 95 °C for 30 s, followed by 40 cycles at 95 °C for 15 s and 60 °C for 1 min. Melting curves were analyzed using the StepOne software. The transcript levels of the genes of interest were normalized to the expression of *EF-1α* and presented as the fold change relative to the corresponding control group at each time point.

### 2.6. In Vivo Experimental Design

Different concentrations of PE were diluted with SSS buffer before being injected. Twelve whiteleg shrimps (7.0 ± 0.4 g in weight) were anesthetized using Eugenol (50 mg/L) (HiMedia Laboratories, Mumbai, India) and injected with 0, 0.08, 0.15, and 0.30 µg PE/g body weight, respectively. The in vivo imaging system (IVIS) Lumina LT series III (Caliper, MA, USA) was used to determine the fluorescence intensities of the PE in the shrimp at 2, 4, 6, and 8 h post-injection through image processing using the appropriate excitation and emission filters [38]. The background signal was subtracted from the two-dimensional images, and the image scaling was established by transforming total radiance efficiency. Fluorescence intensity was described by a multicolor scale ranging from blue (least intense) to red (most intense). Signal intensity figures were superimposed over grayscale reference pictures for anatomical denotation. Scales were set manually to the same values for comparable images to normalize the intensity of the fluorescence across time points. The fluorescence intensity of a specific area of individual samples was measured using the region of interest (ROI) tool in the Live Image 4.5 software (PerkinElmer, Waltham, MA, USA).

### 2.7. Challenge Test

To determine the susceptibility of *L. vannamei* to *V. parahaemolyticus* and WSSV after PE treatment, PE solution was injected into the ventral sinus of the cephalothorax of individual *L. vannamei* (7.0 ± 0.4 g in weight) to achieve doses of 0.08, 0.15, and 0.30 μg PE/g body weight. The challenge test was conducted by injecting 10 μL of bacterial suspension (1 × 10^8^ colony forming units (CFU)/mL) resulting in 1 × 10^6^ CFU/whiteleg shrimp and 9.19 × 10^4^ copies/whiteleg shrimp of WSSV through the ventral sinus of the cephalothorax 24 h after the PE injection. Shrimp that did not receive PE treatment but received the same doses of *V. parahaemolyticus* and WSSV served as the challenge controls. Whiteleg shrimp that received an injection of SSS buffer without either infectious agent served as the unchallenged controls. A total of 110 whiteleg shrimp (10 groups × 11 whiteleg shrimp) were used in this experiment and whiteleg shrimp survival was observed at 12, 24, 36, 48, 60, and 72 h post-challenge. Continuous aeration was provided for each aquarium and 75% of the water was changed every day to remove the impurities and to assure the maintenance of water quality.

### 2.8. Statistical Analysis

The expression of the gene of interest was first normalized to the expression level of housekeeping gene (*EF-1α*). The ratio was then scaled and log2-transformed to improve the normality of the data prior to statistical analysis [39]. A paired sample *t*-test was performed to compare gene expression levels between treatment and control groups with a *p*-value less than 0.05 considered significant. One-way analysis of variance (ANOVA) with Tukey’s post hoc test in the IBM SPSS Statistics Package 22.0 (SPSS Inc., Chicago, IL, USA) was conducted for comparison of the difference between the treatment and control groups in ROS production, PO activity, and phagocytic activity, with a *p*-value less than 0.05 considered significant.

## 3. Results

### 3.1. Purification of Phycoerythrin

**Figure 1:**
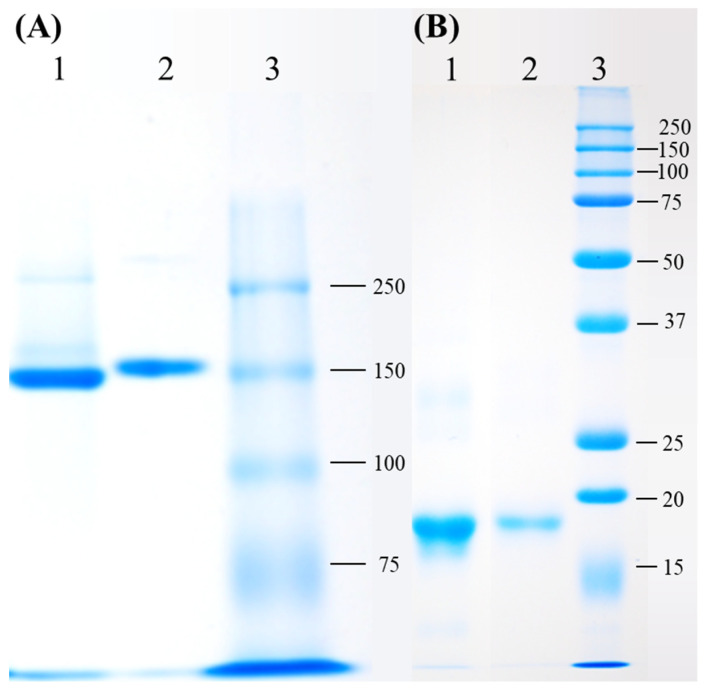
Detection of the purified phycoerythrin (PE) of *Colaconema* sp. via (**A**) native PAGE and (**B**) SDS-PAGE. Native and SDS gels electrophoresis were run in 8% and 15% gel, respectively. (**A**) Native PAGE result. Lane 1, purified PE of *Colaconema* sp.; Lane 2, Standard-PE; Lane 3, protein molecular mass marker. The apparent molecular weight of *Colaconema* sp. was 150 kDa, similar to the standard-PE. (**B**) SDS-PAGE result. Lane 1, purified PE of *Colaconema* sp.; Lane 2, standard-PE; Lane 3, protein molecular mass marker. The apparent molecular weight of *Colaconema* sp. was 15–20 kDa, similar to the standard-PE. The protein in each lane was loaded 5 μg.

**Figure 2:**
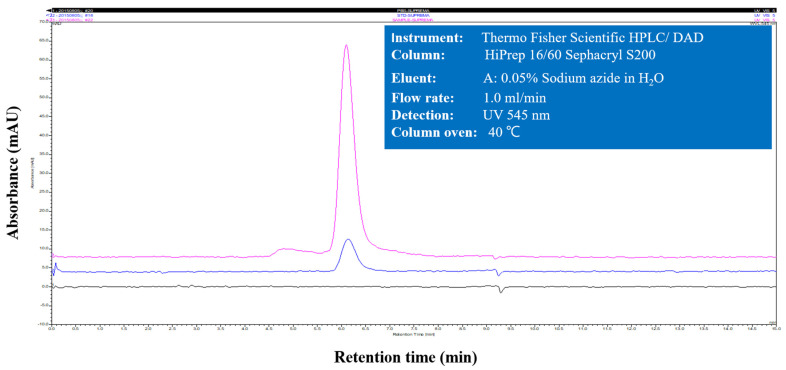
Absorption spectrum of phycoerythrin (PE) purified by size exclusion chromatography in HPLC using a HiPrep Sephacryl S-300 HR column (Pink line: *Colaconema* sp.-PE; blue line: standard-PE; black line: PBS buffer).

**Figure 3:**
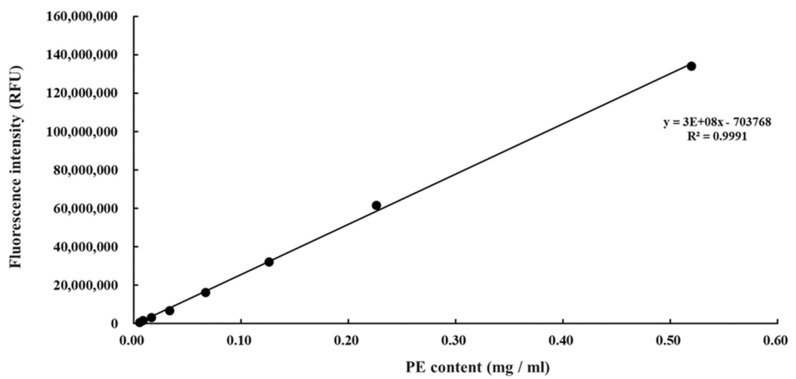
Detection of the fluorescence intensity and phycoerythrin (PE) concentration purified from *Colaconema* sp.

**Figure 4:**
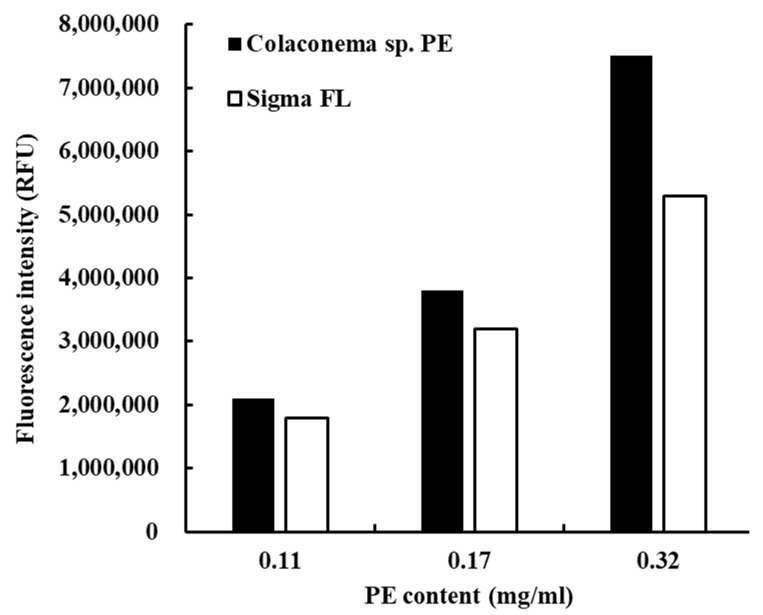
Comparison of the fluorescence intensity of phycoerythrin (PE) purified from *Colaconema* sp. and commercial standard PE (Sigma FL52412) at different concentrations (0.011, 0.017, and 0.032 mg/mL).

An extract of phycobiliproteins was obtained from *Colaconema* sp. and further saturated with ammonium sulfate prior to being applied to a gel filtration column. The purity of the PE from the alga was examined by calculating the ratio of each pigment from the purified PE as well as the purity index. As shown in Table 2, PE, APC, and PC accounted for 93.6%, 4.54%, and 1.8% of the purified proteins, with the purity index at 4.31 for PE. The purified PE protein had a size of 150 kDa in the native PAGE analysis, similar to that of commercial PE (Figure 1A). The subunit components of this fraction were analyzed by SDS-PAGE. There were two bands between 15 and 20 kDa, and a lighter band at 30 kDa (Figure 1B). We compared the purity of *Colaconema* sp. PE to the commercial PE (standard). Our result showed that in 6.5–8 min retention time, the fluorescence emission from the PE extracted in the present study and the commercial standard PE described the same curve (Figure 2). Meanwhile, the light intensity of the PE purified from *Colaconema* sp. increased in a linear dose-dependent manner (Figure 3). Interestingly, at the same concentration, we noticed higher fluorescence intensity in our purified PE than in the commercial PE (Figure 4).

### 3.2. In Vitro Effects of PE on Innate Immune Parameters in Hemocytes

**Figure 5:**
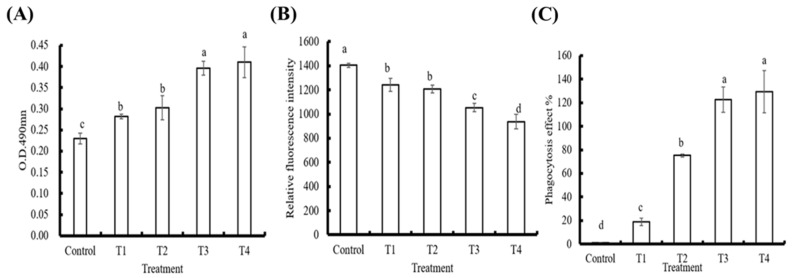
The effects of different phycoerythrin doses on the immune indices of hemocytes. The (**A**) prophenoloxidase activity, (**B**) reactive oxygen species production, and (**C**) phagocytosis activity in hemocytes of whiteleg shrimp (*Litopenaeus* *vannamei*) were examined after treating hemocytes with various doses of purified phycoerythrin (T_1_, T_2_, T_3_, and T_4_ represent treatments of 8, 14, 27, and 52 μg/mL, respectively). Data are presented as the mean ± standard error of mean (*n* = 5). Significant differences (*p* < 0.05, one-way ANOVA and Tukey’s post hoc test) are expressed by different letters. O.D., optical density; RFU, relative fluorescence units.

The effects of the different PE doses on whiteleg shrimp phenoloxidase (PO) activity, ROS, and phagocytosis activity are shown in Figure 5. Compared to the control group, the PO and phagocytosis activity of the hemocytes treated with PE were upregulated in a dose-dependent manner (*p* < 0.05; Figure 5A,C). On the other hand, the ROS production rate was gradually inhibited when hemocytes were treated with increasing doses of PE (Figure 5B).

### 3.3. Observation Using an In Vivo Imaging System after PE Injection

**Figure 6:**
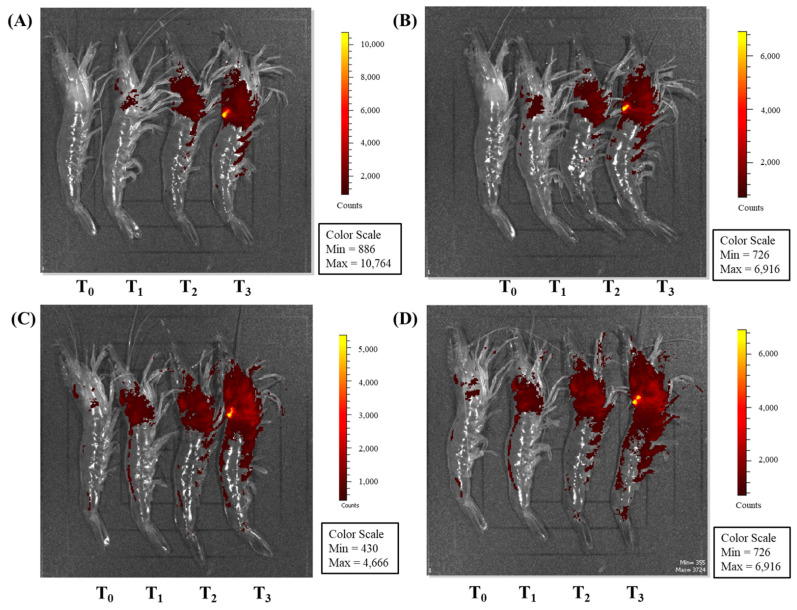
Visualization of whiteleg shrimp after injections of different doses of phycoerythrin. The whiteleg shrimp were observed under an in vivo imaging system (IVIS) at 2 (**A**), 4 (**B**), 6 (**C**), and 8 (**D**) hours post-injection with 0, (T_0_; control), 0.08 (T_1_), 0.15 (T_2_) and 0.30 (T_3_) µg phycoerythrin per gram of whiteleg shrimp body weight.

The visualization of different doses of PE injected into whiteleg shrimp was conducted using an IVIS at 2 h, 4 h, 6 h, and 8 h post-injection. The PE content inside the whiteleg shrimp body was directly related to the injection dose. The signal was observed in the cephalothorax where the PE was injected and gradually diffused to the abdomen and dorsal part of the whiteleg shrimp body (Figure 6).

### 3.4. Expression Analysis of Immune Genes

**Figure 7:**
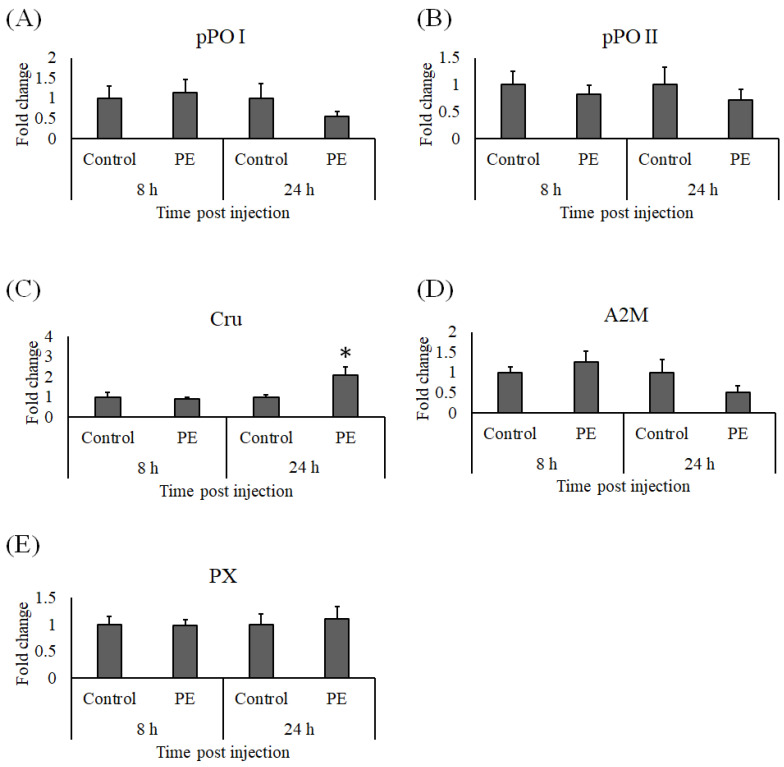
Gene expression of (**A**) *proPO I*, (**B**) *proPO II*, (**C**) *Cru*, (**D**) *A2M*, and (**E**) *PX* in hemocytes from whiteleg shrimp (*Litopenaeus vannamei*) injected with 0.30 μg of phycoerythrin (PE) per gram of whiteleg shrimp body weight or vehicle alone (control). After 8 and 24 h of stimulation, the relative expression levels of immune genes were normalized to the transcript level of *EF1α* and presented as a fold change relative to the control group. Data are shown as the mean ± standard error of the mean (*n* = 5) and significant differences from the control group are denoted by asterisks (*p* < 0.05, paired sample *t*-test).

**Figure 8:**
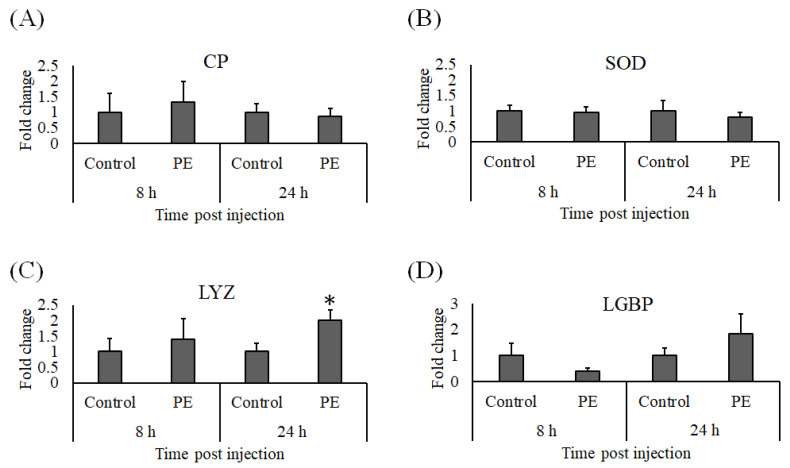
Gene expression of (**A**) *CP*, (**B**) *SOD*, (**C**) *LYZ*, and (**D**) *LGBP* in hemocytes from whiteleg shrimp (*Litopenaeus vannamei*) injected with 0.30 μg of phycoerythrin (PE) per gram of whiteleg shrimp body weight or vehicle alone (control). After 8 and 24 h of stimulation, the relative expression levels of immune genes were normalized to the transcript level of *EF1α* and presented as a fold change relative to the control group. Data are expressed as the mean ± standard error of the mean (*n* = 5) and significant differences from the control group are denoted by asterisks (*p* < 0.05, paired sample *t*-test).

**Figure 9:**
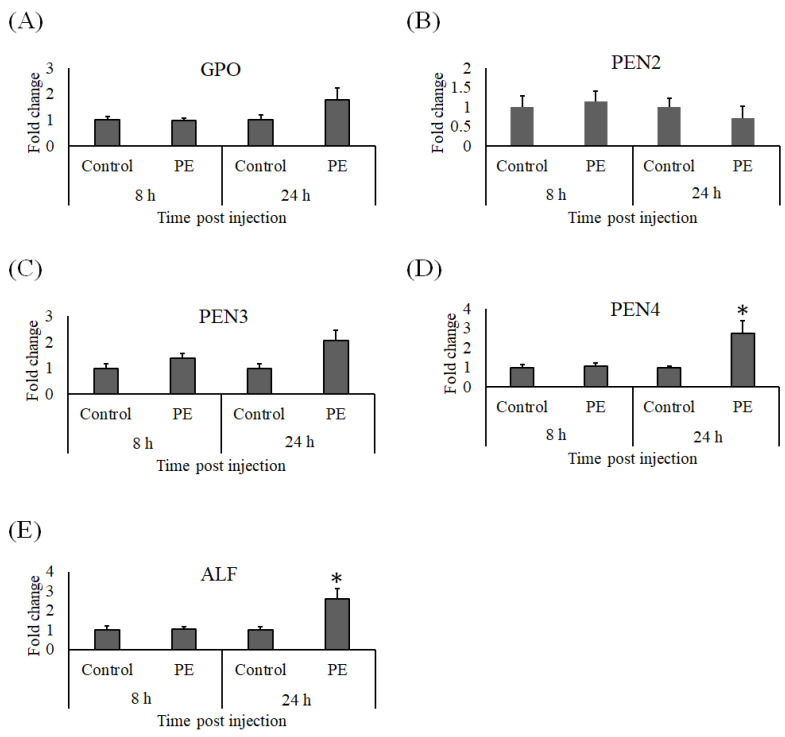
Gene expression of (**A**) *GPO*, (**B**) *PEN2*, (**C**) *PEN3*, (**D**) *PEN4* and (**E**) *ALF* in hemocytes from whiteleg shrimp (*Litopenaeus vannamei*) injected with 0.30 μg of phycoerythrin (PE) per gram of whiteleg shrimp body weight or vehicle alone (control). After 8 and 24 h of stimulation, the relative expression levels of immune genes were normalized to the transcript level of *EF1α* and presented as a fold change relative to the control group. Data are expressed as the mean ± standard error of the mean (*n* = 5) and significant differences from the control group are denoted by asterisks (*p* < 0.05, paired sample *t*-test).

At 8 and 24 h after injection with PE, whiteleg shrimp hemocyte RNA was isolated to detect the effects of PE on the immune response. Genes related to the proPO system (*pPO I* and *pPO II)* were not upregulated by PE injection, and neither were *A2M* or *PX*. However, *Cru* expression was elevated 24 h post PE injection (Figure 7). The expression profiles of *CP* and *SOD* in hemocytes at both sampling times in the PE treated whiteleg shrimp were comparable to the control group (Figure 8A,B), but we noted a significant increase in *LYZ* expression (Figure 8C) and a non-significant induction of *LGBP* 24 h after PE treatment (Figure 8D). Similarly, the expression of *GPO* (Figure 9A) and *PEN3* (Figure 9C) were marginally induced at 24 h post-PE injection, but at the same time point, the transcript levels of *PEN4* and *ALF* were significantly higher in the group that received PE than the group injected with PBS (Figure 9D,E). The *PEN2* transcript levels did not differ significantly between the control and experimental groups (Figure 9B).

### 3.5. Challenge Trials

#### 3.5.1. Effects of PE on the Survival Rate of *L. vannamei* Challenged with *V. parahaemolyticus*

**Figure 10:**
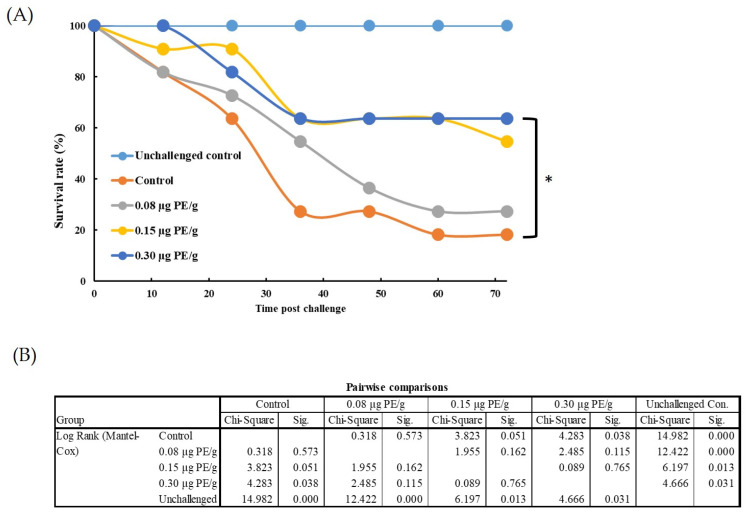
Effects of phycoerythrin (PE) on the survival rate of whiteleg shrimp (*Litopenaeus vannamei*) challenged with *Vibrio parahaemolyticus* during 72 h. The unchallenged control treatment was injected only with whiteleg shrimp saline solution (SSS), while other groups received SSS buffer containing 0, 0.30, 0.15, and 0.08 μg of PE per gram of body weight prior to be challenged with *Vibrio parahaemolyticus* (1 × 10^6^ CFU per shrimp). (**A**) Survivorship curves for whiteleg shrimp after challenged by *V. parahaemolyticus*. (**B**) Differences among the groups were assessed using the Mantel–Cox test for pairwise comparisons. Significant differences (*p* < 0.05) are indicated by asterisks.

As shown in Figure 10, all unchallenged control whiteleg shrimp survived the experiment. However, death began to occur after 12 h in the *V. parahaemolyticus*–challenged whiteleg shrimp. During the period of observation, the survival rates of the whiteleg shrimp that received different doses of PE (0.08, 0.15, and 0.30 μg/g body weight) were always higher than those of the whiteleg shrimp that did not receive PE but were challenged with *V. parahaemolyticus*. The survival rates for whiteleg shrimp that received 0.08, 0.15, and 0.30 μg PE/g body weight prior to the bacterial challenge were 27.27%, 54.55%, and 63.64%, respectively, 72 h post-challenge, while the survival rate was only 18.2% for the challenged control group. The survival rate of whiteleg shrimp was significantly higher in the group that received 0.30 μg PE/g body weight than the untreated control group after exposure to *V. parahaemolyticus.*

#### 3.5.2. Effects of PE on the Survival Rate of *L. vannamei* Challenged with White Spot Syndrome Virus

**Figure 11:**
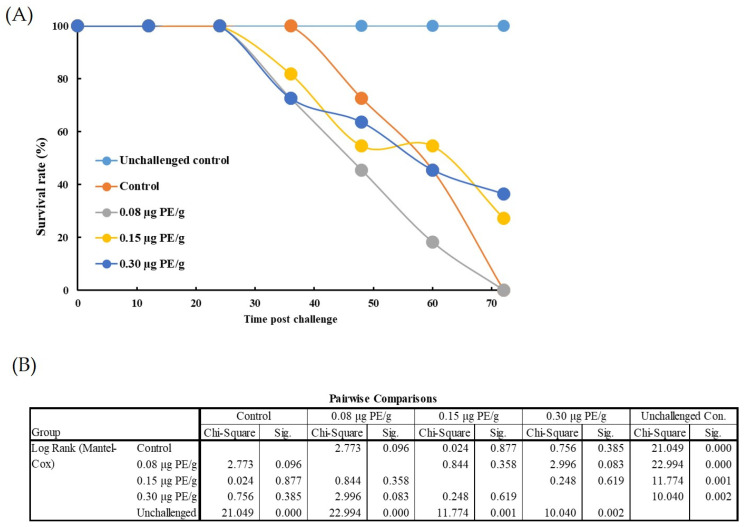
Effects of phycoerythrin (PE) (0, 0.30, 0.15, and 0.08 μg/g body weight) on the survival rate of whiteleg shrimp (*Litopenaeus vannamei*) challenged with whiteleg spot syndrome virus (WSSV) during 72 h. The unchallenged control treatment was injected only with whiteleg shrimp saline solution (SSS), while other groups received SSS buffer containing 0, 0.30, 0.15, and 0.08 μg of PE per gram of body weight prior to be challenged with WSSV (9.19 × 10^4^ copies per shrimp). (**A**) Survivorship curves for whiteleg shrimp after challenged by WSSV. (**B**) Differences among the groups were assessed using the Mantel–Cox test for pairwise comparisons.

In the WSSV challenge trial, death began to occur after 36 h. The survival rates of whiteleg shrimp that received with PE at 0.15 and 0.30 μg/g body weight were insignificantly higher than the survival rate of challenged control whiteleg shrimp 72 h post-challenge (Figure 11). The survival rate at 72 h was 0%, 27.3%, and 36.4% for whiteleg shrimp that had been injected with low, medium, and high doses of PE, respectively, before being treated with WSSV, while no whiteleg shrimp survived in the challenged control group at the end of the observation period. All unchallenged control whiteleg shrimp survived over the experimental period.

## 4. Discussion

Whiteleg shrimp is a crucial food source; its cultural industry plays a key role in human development. Previous studies have demonstrated that multiple terrestrial and marine herbs extract can improve whiteleg shrimp immunity to resist pathogens [40,41,42]. The potential immunomodulatory activity functions of algal PE in mammals have been reported in a previous study [43], but as far as we know, it has not previously been investigated in whiteleg shrimp immunity. Therefore, in this study, we extracted PE from *Colaconema* sp. and tested its effects on whiteleg shrimp immunity.

The proteins extracted and purified from Colaconema sp. had PE as a major component (93.6%) and the molecular weight of PE was verified via gel electrophoresis analysis. A similar molecular weight (~150 kDa) as the commercial standard PE was observed in the native PAGE analysis; however, three bands, instead of the one band expected from the commercial standard PE, were seen in the PE extracted from *Colaconema* sp. when analyzed in SDS-PAGE, suggesting that the PE in *Colaconema* sp. is composed of αβγ subunits, with the most prominent signal between 15 and 20 kDa (Figure 1). Similar results were reported for PE isolated from *Porphyridium cruentum* [44], *Porphyra yezoensis* [45], and *Portieria hornemannii* [46]. We thus conclude that the PE purified in this study has the same subunits as reported in PE sourced from other red algae.

Hemocytes use phagocytosis to directly internalize pathogenic microbes in phagosomes and destroy them using degradative enzymes (e.g., lysozymes) and ROS [47], which are known to destroy bio-macromolecules (e.g., DNA, RNA, proteins, and lipids) through oxidation reactions [48,49]. To avoid excessive ROS that may harm the host itself, SOD is generated to reduce the excessive superoxide anions by transforming them into hydrogen peroxide, to be later degraded by GPO or catalase [50]. ROS production was significantly decreased by PE treatment in vitro (Figure 4), indicating that PE has an anti-oxidation ability that can protect the host from damage by over-enhanced immune responses [51]. Similarly, the expression of *SOD* and *GPO* was not triggered in hemocytes from PE-injected whiteleg shrimp, supporting the notion that PE treatment would not induce (or rather, it would reduce) ROS production; therefore, hemocytes do not need to increase expression of these two antioxidant genes for the conversion of ROS into less toxic substances. Interestingly, the transcript level of *LYZ* was significantly elevated in the whiteleg shrimp injected with PE, suggesting PE treatment could induce certain anti-microbial immune responses.

The proPO cascade is initiated by the binding of pattern-recognition proteins (e.g., lipopolysaccharide- and β-1,3-glucan binding protein (LGBP), C-type lectin, scavenger receptors, galectins, PX, fibrinogen-related proteins, and β-1,3-glucan binding protein (βGBP)) to non-self microbial substances, such as peptidoglycan, β-glucan, and lipopolysaccharide, leading to the activation of the melanization process [52]. PX is one of the pattern recognition proteins that bind microbes and transduces signals to hemocytes through integrins [53,54], and is also involved in cell adhesion and encapsulation-promoting activities [55]. PO activity reveals the status of the innate immune response, including associations with phagocytosis, melanization, and cytotoxic reactions [55,56,57]. In our results, we noticed that PO and phagocytosis activity in PE-treated hemocytes were induced in a dose-dependent manner (Figure 4), suggesting that PE can help to mount immune responses in hemocytes to eliminate invading pathogens, and can also help in wound healing through melanization [58,59,60]. The gene expression analysis did not show upregulation of *LGBP*, *PX*, *proPO I,* or *proPO II,* implying that PE may only affect the proPO system at the protein level. However, we could not exclude the possibility that these genes may be regulated at earlier or later time points after PE injection.

CP and A2M work as antimicrobial molecules, trapping pathogens by hemocyte clotting [61]. A2M can also inactivate harmful proteases [62]. ALF works as an anticoagulant that inhibits the endotoxin-mediated activation of the coagulation cascade [63,64]. Other antimicrobial proteins, such as Cru, ALF, Pen2, Pen3, and Pen4, are known to be important in invertebrates, as they can prevent the spread of pathogens through the blood [65]. Our results show that injection of PE leads to the upregulation of *Cru*, *ALF,* and *Pen4*, but not *Pen2*, *Pen3*, *CP*, or *A2M* in hemocytes. However, the mechanisms underlying the modulation of these genes remain to be determined. Taken together, the injection of PE results in the induction of several immune genes, which may be beneficial to hosts challenged by microbial infection.

To test this hypothesis, we conducted two trials that challenged whiteleg shrimp with *V. parahaemolyticus* or WSSV after injection with SSS buffer or PE. Although injection challenge of whiteleg shrimp is not a natural way of infection, it has been demonstrated to have similar infection performance to naturally infected pathways (e.g., with the nephrocomplex as an entry portal [10]) and reliable reproducibility in indoor systems. In both trials, survival rates were positively related to PE in a dose-dependent manner, supporting the idea that PE could stimulate the immune system of whiteleg shrimp and enhance the survival rate when whiteleg shrimp are faced with either viral and bacterial pathogens.

## 5. Conclusions

This study revealed that a macroalgae (*Colaconema* sp.) source of PE has immune-regulatory potential, supported by the evidence of the raising of immune parameters and gene expression. Additionally, injections of PE improved the survival rate of whiteleg shrimp after infection by WSSV or *V. parahaemolyticus*.

## Figures and Tables

**Table 1:** Primers used in this study.

Gene	Sequence 5′–3′	Reference
Lipopolysaccharide and β-1,3-glucan binding protein (*LGBP*)	CGGCAACCAGTACGGAGGAAC	(Cheng et al., 2005) [34]
	GTGGAAATCATCGGCGAAGGAG	
Peroxinectin (*PX*)	ATCCAGCAGCCAGGTATG	(Liu et al., 2004) [35]
	CAGACTCATCAGATCCATTCC	
Prophenoloxidase I (*proPO I*)	ACGTCACTTCCGGCAAGCGA	(Chen et al., 2014) [36]
	CCTCCTTGTGAGCGTTGTCAGG	
Prophenoloxidase II (*proPO II*)	ACCACTGGCACTGGCACCTCGTCTA	
	TCGCCAGTTCTCGAGCTTCTGCAC	
α2-macroglobulin (*A2M*)	GCACGTAATCAAGATCCG	
	CCCATCTCATTAGCACAAAC	
Anti-lipopolysaccharide factor (*ALF*)	CTGTGGAGGAACGAGGAGAC	(Wang et al., 2010) [37]
	CCACCGCTTAGCATCTTGTT	
Crustin (*Cru*)	GAGGGTCAAGCCTACTGCTG	
	ACTTATCGAGGCCAGCACAC	
Penaeidin 2 (*Pen2*)	TCGTGGTCTGCCTGGTCTT	
	CAGGTCTGAACGGTGGTCTTC	
Penaeidin 3 (*Pen3*)	CACCCTTCGTGAGACCTTTG	
	AATATCCCTTTCCCACGTGAC	
Penaeidin 4 (*Pen4*)	GCCCGTTACCCAAACCATC	
	CCGTATCTGAAGCAGCAAAGTC	
Lysozyme (*Lyz*)	GAAGCGACTACGGCAAGAAC	
	AACCGTGAGACCAGCACTCT	
Superoxidase dismutase (*SOD*)	ATCCACCACACAAAGCATCA	
	AGCTCTCGTCAATGGCTTGT	
Glutathione peroxidase (*GPO*)	TTTTTCCGTGCAAAAAGGAC	
	TAATACGCGATGCCCCTAAC	
Clotting protein (*CP*)	TCTTTGCGCAGTTGGTGATC	
	TGAGGTGACCGAGTGCAAAA	
Elongation factor 1α (*EF1α*)	ATGGTTGTCAACTTTGCCCC	(Chen et al., 2014) [36]
	TTGACCTCCTTGATCACACC	

**Table 2:** The ratio in phycoerythrin (PE), allophycocyanin (APC), and phycocyanin (PC) concentration of purified pigments of *Colaconema* sp.

Pigments Source	Phycoerythrin	Allophycocyanin	Phycocyanin
*Colaconema* sp.	93.6%	4.54%	1.8%

## Data Availability

The authors confirm that the data supporting the findings of this study are available within the article.
